# Developmental subgroups and co-occurring conditions among 5817 minimally speaking autistic youth: a latent class analysis of the SPARK cohort

**DOI:** 10.21203/rs.3.rs-11381780/v1

**Published:** 2026-11-01

**Authors:** Rachel Reetzke, Vini Singh, Diogo Fortes, Elizabeth Eiler, Calliope Holingue

**Affiliations:** Kennedy Krieger Institute; Kennedy Krieger Institute; Johns Hopkins Bloomberg School of Public Health; Kennedy Krieger Institute; Kennedy Krieger Institute

**Keywords:** Autism, Minimally speaking, Latent class analysis, Co-occurring conditions, Motor development, Nonverbal cognition, Social interaction

## Abstract

**Background:**

Approximately 30% of autistic children remain minimally speaking beyond kindergarten, using few or no spoken words. Although often studied as a single group, these children show a wide range of social, motor, and nonverbal cognitive abilities. Few studies have systematically examined this heterogeneity. Characterizing minimally speaking autistic subgroups is an important first step toward understanding the genetic and neurobiological mechanisms underlying different spoken language outcomes in autism.

**Methods:**

To fill this gap, we conducted a cross-sectional study of 5817 minimally speaking autistic youth aged 5–17 years (76.8% male) who participated in the Simons Powering Autism Research for Knowledge national study. We estimated latent class models separately for children (5–11 years, n = 4682) and adolescents (12–17 years, n = 1135) using four indicators: everyday spoken language level, social interaction difficulty, number of delayed early motor milestones, and nonverbal cognitive ability. We compared classes on sociodemographic characteristics, co-occurring conditions, and total interventions received using chi-square, Fisher exact, and Welch tests with false discovery rate correction.

**Results:**

We identified three child subgroups and two adolescent subgroups, ranging from lower to higher skills across all four domains. Strengths in one domain generally accompanied strengths in others. Within subgroups, early motor development was a relative strength in lower-skill groups and social interaction in higher-skill groups. Among children, lower-skill subgroups had a higher prevalence of atypical head growth, seizures or epilepsy, obsessive-compulsive disorder, sleep disorders, and feeding or eating disorders (sleep disorder, higher- vs intermediate-skill child subgroup: odds ratio 0.54, 95% confidence interval (CI) 0.46–0.63) and also received more interventions (lower- vs higher-skill child subgroup: 1.9 more, 95% CI 1.7–2.2).

**Limitations:**

All latent class indicators were derived from caregiver-report questionnaires, which may be subject to recall bias. Although our results show high split-half agreement of class assignments, they still need to be replicated in an independent cohort of autistic children.

**Conclusions:**

Minimally speaking autistic youth show clinically meaningful heterogeneity in their developmental characteristics, co-occurring conditions, and service use. Future studies should examine the extent to which underlying genetic etiologies contribute to these subgroup differences.

## Background

Approximately 30% of autistic children are minimally speaking, using few or no spoken words beyond kindergarten [1–3]. The term “minimally speaking” (also referred to as “minimally verbal”) has been used to describe the spoken language abilities of a heterogeneous group of autistic children, ranging from those who produce no spoken language to those who use some words or specific phrases that are restricted in context, frequency, or function [4, 5]. Historically, minimally speaking autistic children have been excluded from autism research. This is partially due to barriers in completing required standardized assessments, which often require overt verbal responses [1, 5]. As a result, our current evidence base may not adequately reflect the abilities and needs of minimally speaking autistic youth [1, 6, 7].

Developmental cascades and dynamic systems theories posit that language both emerges from and shapes development in other domains, including social interaction, motor skills, and nonverbal cognition [8–11]. Consistent with this view, early gross and fine motor skills have been found to be significantly associated with spoken language abilities in autistic children [12, 13]. For example, one study found that fine motor skills were associated with greater speech intelligibility in a group of minimally speaking autistic children aged 4 to 7 years [12]. Another large-scale study of autistic children with severe language delays found that social interaction and cognitive ability were predictive of phrase and fluent speech attainment [14]. At the same time, children with similarly limited speech have shown significant heterogeneity in nonverbal cognitive abilities. For example, in a Simons Simplex Collection study, 16% of children classified as minimally verbal had nonverbal IQ scores of 70 or higher, and approximately half had verbal abilities substantially below their estimated nonverbal cognitive ability [5]. This suggests that limited spoken language ability may arise from different developmental pathways. Characterizing subgroups across multiple developmental domains may therefore help elucidate different pathways to limited use of spoken language in autism.

Two data-driven studies started to address this gap. In one study, a latent profile analysis revealed three subgroups within 344 minimally speaking autistic children aged 3 to 8 years. These groups included a large, globally delayed group; a group with stronger nonverbal cognition, receptive language, and social communication skills but limited spoken language; and a smaller group with intermediate nonverbal cognition and the highest number and variety of spoken words [15]. Another pre-registered cluster analysis of a transdiagnostic cohort of 193 minimally speaking school-age children, in which an autism diagnosis was not required, identified six clusters among 181 children with complete clustering data [16]. Four of the six clusters reflected very low-, low-, mid-, or high-skill profiles across receptive, expressive, adaptive, and motor skills. Two reflected discrepant profiles in which spoken language abilities were disproportionately lower than receptive and broader communication skills. Clusters also differed in diagnostic profile, use of augmentative and alternative communication, and echolalia. Together, these findings suggest that there may be distinct developmental subgroups of minimally speaking autistic children. However, only one of the two studies focused exclusively on minimally speaking autistic children. As a result, evidence on developmental subgroups specific to minimally speaking autistic youth remains limited.

Minimally speaking autistic youth also present with myriad co-occurring conditions. Sleep difficulties and epilepsy are common co-occurring conditions in autism, and epilepsy is particularly frequent among autistic individuals with intellectual disability [17]. Macrocephaly and brain overgrowth have also been reported in subsets of autistic individuals [18]. Mental health assessment may be affected by diagnostic overshadowing, in which psychological symptoms are misattributed to intellectual disability, although evidence for this potential bias is mixed [19]. In addition, families of autistic children have reported unmet needs for intervention services [20], and relatively few interventions have been developed and tested specifically for minimally speaking autistic children [7]. Little is known about whether co-occurring conditions and service use differ systematically among specific developmental subgroups of minimally speaking youth.

## The present study

While previous studies have advanced our understanding of developmental heterogeneity present in minimally speaking autistic children [5, 15, 16], they used smaller samples with varying age ranges and inclusion criteria. Therefore, it is unclear whether profiles generalize to the broader autism population or to adolescents. In addition, profiles have been largely defined by communication measures rather than by developmental domains that shape language development. Finally, few studies have examined whether identified subgroups can be further distinguished by co-occurring conditions and service use [15, 16, 21].

The present study addresses these gaps using data from the Simons Powering Autism Research for Knowledge (SPARK) study, a large, geographically diverse autism cohort in the United States [22, 23]. SPARK enrolls autistic individuals of all ages and ability levels, making it possible to study thousands of minimally speaking children and adolescents who are often underrepresented in research that relies on direct standardized testing. Leveraging SPARK, our first aim was to identify developmental subgroups among minimally speaking children (5–11 years) and adolescents (12–17 years) using latent class analysis. Guided by the developmental cascades framework, we selected four primary indicators for our latent class analyses that have been found to vary among minimally speaking children [5, 15, 16]: spoken language level, social interaction, early motor milestone delays, and nonverbal cognitive level [12–14]. All four indicators were caregiver-reported and therefore available regardless of a child’s spoken language level. Our second aim was to compare identified latent classes on variables not used to derive them [24], including sociodemographic characteristics, co-occurring conditions, and service use. Based on prior work [15, 16], we hypothesized that distinct minimally speaking subgroups would emerge and that these subgroups would differ significantly in co-occurring conditions and service use.

## Methods

The Johns Hopkins Medicine Institutional Review Board approved this secondary data analysis (IRB00460006). This study followed the Strengthening the Reporting of Observational Studies in Epidemiology (STROBE) reporting guideline (Additional file 2) [25].

### SPARK

This cross-sectional study received permission to use de-identified data from the SPARK Phenotype Dataset (version 16, released July 2025) [22, 23]. SPARK is the largest autism cohort in the United States. Families enrolled in SPARK online or through collaborating clinical sites. Caregivers provided consent and completed questionnaires spanning family and medical history, developmental and treatment history, and standardized measures.

### Participants

No a priori sample size calculation was performed. Instead, sample size was determined by the number of eligible SPARK participants. Our analytic sample included children 5 to 17 years of age with a parent-reported professional autism diagnosis. Minimally speaking was defined as everyday spoken language of no words, single words, or three-word phrases. We selected this criterion to capture a range of limited verbal abilities in our age range, including minimally speaking school-age children and adolescents who may use echolalic or scripted speech to communicate [4].

All participants were required to have the following parent-report measures completed: SPARK Registration Questionnaire, Background and History Questionnaire-Child/Dependent (BHQ), and Social Communication Questionnaire-Lifetime (SCQ-L) within 6 months of their SPARK registration. Children using longer flexible sentences, not meeting SCQ-L validity criteria, or missing any indicator were excluded.

The complete-case sample comprised 5817 participants (Figure S1 and Table S1 in Additional file 1). Of 6311 eligible children and adolescents, 494 (7.8%) were excluded because of missing motor or nonverbal cognition data (see Table S2 for a comparison of included and excluded participants). Data on co-occurring conditions were available for 5802 (99.7%) participants, and intervention data were available for all 5817.

### Latent Class Indicators

Our indicators were selected a priori to capture cross-domain developmental variation relevant to communication [12–14]. The selection of domains was guided by the developmental cascades framework, which posits that language, social communication, motor development, and nonverbal cognitive ability are interrelated [8–11]. These same domains of development are also commonly prioritized by clinicians when assessing minimally speaking autistic youth [26, 27]. Sources of measurement and justification for the selection of each indicator can be found in Table S3.

### Spoken Language

Everyday spoken language (parent-report SPARK Registration Questionnaire) was an ordinal indicator with three levels (no words, single words used meaningfully, or three-word phrases). This item was strongly associated with the SCQ-L phrase item (polychoric correlation, 0.93).

### Social Interaction

Social interaction was assessed using the 20-item SCQ-L social interaction domain (range, 0–20, with higher scores indicating greater difficulty) [28]. Records missing SCQ-L item 1 or at least two social interaction items were excluded. For participants with one missing social interaction item, the domain score was the sum of available items without proration. This domain combines historical and current or lifetime behavioral items. The commonly used SCQ-L total-score screening cutoff of 15 does not apply to this domain score.

### Motor Delays

Consistent with previous studies, motor delays were counted across four caregiver-reported milestones used to characterize developmental variability in autism [29]: sitting without support, crawling, walking, and feeding oneself with a spoon. We coded attainment after 8, 12, 16, and 24 months, respectively, or “not yet” as delayed (range, 0–4). The milestone-delay count was associated with a caregiver-reported professional motor disorder diagnosis on Basic Medical Screening (two-step polychoric correlation, 0.52; n = 5802; Table S3).

### Nonverbal Cognition

Nonverbal cognition was estimated using an item from the parent-report BHQ. The item asked caregivers to estimate their child’s nonverbal problem-solving ability relative to chronological age (at or above, slightly below, or significantly below age). Comparisons with reported intellectual disability and neuropsychological cognitive testing results in a subset of participants are described in Table S3.

## Clinical Characteristics of Minimally Speaking Subgroups

### Sociodemographic Characteristics

Sociodemographic characteristics included child sex, race and ethnicity, household income, parental education and occupation, and age at registration and at autism diagnosis. Race and ethnicity were reported by caregivers during SPARK registration using fixed categories. These variables were used to characterize the sample and evaluate the generalizability of findings.

### Co-occurring Conditions

Co-occurring conditions were parent-reported lifetime professional diagnoses (Basic Medical Screening). Basic Medical Screening questionnaires used in this analysis were completed during 2017–2025. We grouped conditions as neurodevelopmental and medical (e.g., seizures or epilepsy, birth defects); mental health and behavioral (e.g., anxiety, obsessive-compulsive disorder, attention-deficit/hyperactivity disorder); and functional (sleep, feeding or eating). Endorsed diagnoses were coded as present and unselected checkboxes on matched forms as absent. Condition groupings were adapted from Litman et al. [30].

### Intervention and Service Use

Service use was the total number of endorsed school-based and private or community service items ever received (BHQ; full list in Supplementary Methods 1). The same modality could be counted in both settings.

## Data Analysis

### Latent Class Analysis

Latent classes were identified using finite mixture models applied to one continuous indicator (SCQ-L social interaction domain) and three ordinal indicators (spoken language level, number of motor delays, and level of nonverbal cognition). Because our domains of interest change throughout development [31–36], we stratified the sample into child (5 to 11 years) and adolescent (12 to 17 years) groups and modeled each separately. Models were fit with the clustMD package [37] across covariance parameterizations, comparing two to nine classes for children (Figure S2) and two to seven classes for adolescents, as models with more classes did not converge (Figure S3). The equal-volume, varying-shape (EVI) specification had the lowest BIC in both age groups [38]. We selected the solution based on the Bayesian information criterion (BIC) [39], the integrated completed likelihood (ICL) [40], a 5% minimum class size, and clinical interpretability (Supplementary Methods 2, Figures S2 and S3, and Table S4).

We assessed classification certainty using the saved mixture-model posterior probabilities (relative entropy and mean maximum posterior probability), supplemented by a post hoc local-independence classifier. We also assessed split-half agreement of classifier assignments (adjusted Rand index) [41], conditional on the full-sample latent class assignments (Supplementary Methods 2). In an alternative model, we added child age as a fifth, continuous indicator (Supplementary Methods 3, Figure S4, and Table S5). That model primarily distinguished the cohort by age rather than by developmental profile. The primary analyses therefore used separate models for children and adolescents, each with only the four a priori selected developmental indicators. Co-occurring conditions and service use were examined after model selection and were not used to select the final number of classes. Exploratory indicator-specific analyses used the final fitted parameters to calculate univariate relative entropy and the change in entropy after omitting each indicator from posterior calculation while holding all parameters fixed (Supplementary Methods 4 and Table S6).

### Subgroup Characteristics

Because classes were estimated separately by age group, all class comparisons (both omnibus and post hoc) were conducted within each age group. Omnibus differences across classes were tested with Welch one-way analysis of variance (continuous) [42] and the Pearson chi-square or Fisher exact test (categorical). Effect sizes were eta-squared or bias-corrected Cramér V [43], and condition prevalences were reported with 95% Wilson score confidence intervals [44]. Post hoc pairwise comparisons were reported as odds ratios (Fisher exact test) for co-occurring conditions and mean differences with 95% confidence intervals for intervention counts (Welch t tests). We controlled the false discovery rate with the Benjamini-Hochberg procedure [45], separately within each age group, and report adjusted P values as q values. For omnibus tests, P values were adjusted across the 12 co-occurring condition and service-use outcomes and, separately, across the sociodemographic characteristics. For post hoc tests in children, P values were adjusted across the three pairwise class comparisons for each outcome. For adolescents, statistical inference and figure annotations use omnibus q values adjusted across the 12 outcomes. Supplementary pairwise Fisher and Welch tests compare the same two classes but report unadjusted P values. Analyses were conducted in R versions 4.4.0, 4.5.3, and 4.6.1 (packages: tidyverse, gtsummary, rstatix, effectsize, nlme, rcompanion, Hmisc, and polycor). A q value less than .05 was considered significant.

## Results

### Latent Models

For children, the three-class solution had a lower BIC than the two-class solution (44548.4 vs 44620.7). In contrast, ICL and classification certainty favored the two-class solution. Model-based relative entropy was 0.50 for three classes versus 0.69 for two classes (mean maximum posterior probability, 0.75 vs 0.91). We selected the three-class solution, which kept every class above the 5% minimum. This prioritized a clinically meaningful solution over a two-class solution with more distinct class separation (Supplementary Methods 2 and Table S4). Among children, the largest class had relatively higher skills across the indicators (higher-skill class 2; 57%). Two smaller classes had intermediate-skill (class 1; 24%) and lower-skill (class 3; 19%) profiles, with less spoken language, more motor delays, greater difficulty with social interaction, and a lower level of nonverbal cognitive ability (Table 1). Among adolescents, lower-skill (class 1; 48%) and higher-skill (class 2; 52%) profiles were identified. Model-based entropy was 0.54 and mean maximum posterior probability was 0.85. Figure 1 shows the primary three-class child and two-class adolescent profiles across all four indicators. Agreement between held-out classifier assignments and the full-sample class assignments averaged adjusted Rand indices of 0.84 for children and 0.93 for adolescents.

In our exploratory indicator-specific analyses, social interaction provided the greatest univariate class separation (entropy: children, 0.386; adolescents, 0.481), followed by spoken language (0.221; 0.075), nonverbal cognition (0.193; 0.066), and motor delays (0.139; 0.033). Omitting social interaction from posterior calculation reduced entropy most (0.178 and 0.370), whereas omitting motor delays reduced it least (0.016 and 0.008; Table S6).

### Within-Class Developmental Profiles

Within each class, scores differed across developmental domains (Friedman P < .001; Supplementary Methods 5 and Tables S7 and S8). In the lower-skill classes (child class 3; adolescent class 1), motor milestone delays were the least elevated of the four standardized domain scores. This is a relative pattern within each class. The lower-skill classes still exhibited more motor delays than the higher-skill classes: at least one milestone was delayed in 76% versus 42% of children and 62% versus 39% of adolescents (Table 1). In the higher-skill classes (class 2 in both age groups), social interaction was the relative strength.

These within-class patterns describe relative strengths and difficulties. They do not reflect absolute levels of development. A higher-skill profile did not indicate age-typical development. For example, in the higher-skill child class (class 2), 63% were rated below age level in nonverbal cognition, 42% had at least one motor milestone delay, and 27% were reported to use either no words or single words. Caregiver ratings of nonverbal cognition at or above age level were concentrated in, but not limited to, the higher-skill classes. Of the 1119 children with this rating, 1003 (89.6%) were in the higher-skill class, 82 (7.3%) in the intermediate-skill class, and 34 (3.0%) in the lower-skill class. Of the 111 adolescents with this rating, 91 (82.0%) were in the higher-skill class and 20 (18.0%) in the lower-skill class.

### Subgroup Characteristics

There were small but statistically significant differences in sociodemographic characteristics across classes (Table S9). Specifically, among children, classes differed in age at registration and age at diagnosis (both q < .001). The subgroup with lower skills (class 3) was diagnosed earliest but registered in the SPARK study relatively later. Child classes also differed in race (q = .04), maternal education (q = .03), and maternal occupation (q < .001). However, effect sizes were small (bias-corrected Cramér V, 0.03–0.06). Child classes did not significantly differ in household income (q = .42). Among adolescents, only age at diagnosis was significantly different (q < .001), with the lower-skill class diagnosed earlier than the higher-skill class (mean, 41.6 vs 53.4 months). Across all sociodemographic comparisons, effect sizes were small (all η^2^ < 0.05, all bias-corrected Cramér V < 0.09).

Co-occurring conditions and service use significantly differed across subgroups (Table 2, Fig. 2, and Table S10). Among children, prevalence differed across classes (all q < .05) for macro- or microcephaly, seizures or epilepsy, birth defects, obsessive-compulsive disorder, and sleep and feeding or eating disorders. The largest effect sizes were reported for sleep (Cramér V = 0.16) and feeding or eating (0.15). Tics, depression, anxiety, attention-deficit/hyperactivity disorder, and conduct or aggression did not significantly differ by group. In post hoc comparisons, the higher-skill class had lower odds of atypical head growth, seizures or epilepsy, birth defects, obsessive-compulsive disorder, sleep disorders, and feeding or eating disorders compared with both the intermediate-skill and lower-skill classes (e.g., sleep, class 2 vs 1: odds ratio, 0.54 [95% CI, 0.46–0.63]). The intermediate-skill child class also differed from the lower-skill class in sleep and feeding or eating diagnoses (q = .007 and .03, respectively).

Fewer between-class differences were statistically significant among adolescents. Sleep disorders (q = .003) and feeding or eating disorders (q = .04) were more prevalent in the lower-skill class. Service use showed a similar pattern, with the lower-skill child class receiving more interventions than the higher-skill class (class 3 vs 2: mean difference, 1.9 interventions [95% CI, 1.7–2.2]), and a smaller difference among adolescents (class 1 vs 2: mean difference, 0.73 interventions [95% CI, 0.25–1.22]). Mean intervention counts increased from 8.1 in the higher-skill class to 9.1 in the intermediate-skill class and 10.0 in the lower-skill class. All pairwise child comparisons of intervention counts had q < .001.

## Discussion

To our knowledge, this is the largest study to identify developmental subgroups among minimally speaking autistic youth, a group that has historically been understudied. Using a data-driven approach, we identified three classes among minimally speaking autistic children (5–11 years) and two among minimally speaking autistic adolescents (12–17 years). In both age bands, classes ranged from lower to higher skill levels, with strengths in one developmental domain often accompanying strengths in the others. Within subgroups, however, we observed some variation across developmental domains. Sociodemographic differences between classes were small. In contrast, subgroups significantly differed in their presentation of co-occurring conditions and service use.

Our results align with and extend prior efforts to characterize heterogeneity among minimally speaking autistic children, using a much larger sample recruited across the United States [15, 16]. First, consistent with Pizzano et al. [15], subgroups differed in overall developmental level. Classes with less spoken language also showed more motor delays, greater social interaction difficulties, and lower nonverbal cognition. This result is consistent with a developmental cascades framework in which early differences in one domain are associated with differences in others [8]. At the same time, consistent with Saul et al. [16], who identified two subgroups in which spoken language was disproportionately lower than other abilities, profiles were not uniform across domains. Motor development appeared to be a relative strength in the lower-skill subgroups. In contrast, social interaction was a relative strength within the higher-skill subgroups. We assessed basic, early-emerging motor milestones (i.e., sitting, crawling, walking, and self-feeding), which may be relatively preserved even when later-developing language and cognitive skills are more affected. In addition, our indicator predominantly captured early gross motor milestones and did not directly assess fine motor skills, which have been associated with expressive language in minimally speaking children [12]. Direct fine motor assessment is needed to determine whether this relative strength extends to fine motor skills.

Fewer classes emerged among adolescents. This finding may reflect factors related to development, cohort effects, or recall bias. Many autistic children who are minimally speaking or have early language delays go on to develop spoken language [1]. As such, those who remain minimally speaking into adolescence may represent a more homogeneous subgroup [14]. This pattern may also reflect cohort differences between adolescents and younger children in SPARK. Changes in diagnostic and reporting practices over time may also have contributed to differences between age groups [46]. Given that most participants registered in SPARK between 2017 and 2019, adolescents were born and diagnosed several years earlier than children, often before the 2013 introduction of the Diagnostic and Statistical Manual of Mental Disorders, Fifth Edition (DSM-5) criteria. Autism screening, awareness, and acceptance have also significantly expanded over the past 10 years. Adolescents who were diagnosed during that earlier period may therefore represent a more homogeneous group with more similar developmental profiles and higher support needs. Consistent with this, adolescents in our sample received their autism diagnosis later than children (mean, 47.7 vs 43.4 months), and more were rated by caregivers as significantly below age level in nonverbal cognition (73.0% vs 48.0%; Table S1). It is also possible that parent-reported indicators are less sensitive at older ages, because caregivers must recall milestones from a decade or more earlier [47]. Future work can test these hypotheses by prospectively following minimally speaking autistic children from childhood into adolescence.

Among children, co-occurring conditions and service use followed two patterns. First, neurodevelopmental and medical conditions (atypical head growth, seizures or epilepsy, and birth defects) were less common in the higher-skill class than in the intermediate- and lower-skill classes, which did not differ from each other. The second pattern we observed was that sleep and feeding or eating disorders and total interventions increased stepwise from the higher- to the intermediate- to the lower-skill class, and all three were also higher in the lower-skill adolescent class. These findings align with reports of frequent sleep difficulties in autism, as well as a high occurrence of epilepsy among autistic individuals with intellectual disability [17]. In addition, they align with literature on atypical head growth in autism. For example, in a study of more than 1800 autism families, IQ decreased as head circumference deviated from the size expected (based on parent head size) [48]. Microcephaly in particular has been linked to lower cognitive ability and seizures [49]. From a clinical standpoint, routine screening for sleep, feeding, and seizure concerns may be warranted for all minimally speaking autistic youth during well-child visits. Etiologically, the co-occurrence of atypical head growth, seizures, and birth defects in the intermediate- and lower-skill subgroups raises the question of whether these subgroups share underlying genetic contributions.

Both atypical head growth and seizures are hallmark features of several single-gene forms of autism. For example, in one exome sequencing study of autistic children with microcephaly or macrocephaly, approximately one quarter carried a pathogenic or likely pathogenic variant, most in genes known to regulate head size [50]. At the macrocephaly end, disruptive variants in CHD8, a chromatin remodeler, are associated with brain overgrowth and intellectual disability [51, 52]. At the microcephaly end, loss-of-function variants in DYRK1A, which regulates neuronal proliferation, cause a syndrome of microcephaly, intellectual disability, impaired speech, feeding difficulties, and seizures [53, 54]. In SPARK, autistic individuals with loss-of-function variants in highly penetrant genes have been found to be more likely to have cognitive impairment than those with variants in moderate-risk genes [55]. Recently, data-driven identified phenotypic classes in the SPARK cohort have been found to map onto distinct patterns of de novo, inherited, and common genetic variation [30]. Together, these findings suggest that lower-skill minimally speaking subgroups may be enriched for rare, high-impact genetic variants, a hypothesis that can be tested directly using SPARK’s genetic data.

Patterns among mental health conditions were less consistent. Obsessive-compulsive disorder was more common in the intermediate- and lower-skill child subgroups, whereas the prevalence of anxiety and depression was not significantly different across classes within either age group. Overlap between repetitive behaviors in autism and obsessive-compulsive disorder can complicate differential diagnosis [56]. Descriptively, reported anxiety and depression diagnoses were more common among adolescents than among younger children. It should be noted that these cross-sectional differences cannot distinguish age-related prevalence from differences in recognition or reporting. Diagnostic overshadowing is one possible assessment concern in people with intellectual disability [19] but was not measured here. Finally, greater service use in the lower-skill subgroups may reflect greater need. However, intervention counts do not establish whether needs were met or the quality of services received. Evidence indicates that caregivers of autistic children report unmet specialty and therapy needs [20], supporting future assessment of service access, quality, and fit with individual needs.

### Implications for Clinical Practice

These profiles may inform future intervention planning for minimally speaking autistic youth. Because subgroups differed across motor, social-interaction, and nonverbal-cognitive domains rather than by spoken language alone, clinical care should focus on multiple developmental domains, not solely on spoken language [6]. Profile membership should not prescribe a communication modality, as communication needs are more complex than word count [57]. Assessment should consider the child’s communication across modalities, including augmentative and alternative communication, and identify accessible ways to communicate [6]. In addition, our findings may help clinicians ask which motor, cognitive, and social interaction skills could be leveraged to support a child’s use of augmentative and alternative communication. Beyond communication, youth with higher support needs may warrant proactive screening for sleep and feeding disorders, conditions that can be missed when spoken language is limited.

### Implications for Future Research

Our findings complement recent SPARK work in which broad phenotypic classes of autistic individuals, defined across core features, developmental milestones, and co-occurring conditions, were associated with distinct patterns of common, de novo, and rare inherited genetic variation [30]. The developmental subgroups identified here provide additional phenotypic profiles to test whether genetic variation also differs significantly among minimally speaking youth.

Minimally speaking autistic youth are often underrepresented in research [1, 5, 6]. Caregiver-report approaches can broaden participation but should be complemented by accessible direct assessment. Combining inclusive recruitment and assessment with person-centered analyses has the potential to characterize variation without treating limited spoken language as a complete description of communication [57]. Whether the identified subgroups differ in their response to certain interventions over others should also be tested prospectively.

## Limitations

All indicators, diagnoses, and service-use variables were reported by caregivers, and no direct standardized assessments or measures of augmentative and alternative communication use were available. Caregivers recalled motor milestone ages, in some cases a decade or more later, and rated nonverbal cognitive level relative to age. The SCQ-L social interaction domain also combines historical and current or lifetime items, so it does not capture current social interaction alone. Recall error and changes in behavior over time may therefore have affected profiles, especially in adolescents, and complicate comparisons with contemporaneous language and cognition ratings. In addition, SPARK is a volunteer cohort, and our analytic sample was predominantly White (67.2% of those with race data) and male (76.8%). The subgroups and prevalence estimates may therefore not generalize to all minimally speaking autistic youth, particularly those from communities underrepresented in research. The direction and magnitude of any bias in subgroup profiles and condition estimates from caregiver reporting, volunteer recruitment, and exclusion of participants with missing indicators remain uncertain. Finally, split-half checks showed high agreement of classifier assignments (adjusted Rand index, 0.84 for children and 0.93 for adolescents), but these checks were conditional on the full-sample latent classes and do not replace external validation. We did not have access to an independent cohort of minimally speaking autistic youth with comparable measures across all four developmental domains, so replication in such a cohort is an important next step.

## Conclusions

Our findings indicate that minimally speaking autistic youth show clinically meaningful heterogeneity in their developmental characteristics, co-occurring conditions, and service use. We identified developmental subgroups, and lower-skill child subgroups had a higher prevalence of co-occurring conditions, including atypical head growth and seizures, and received more interventions. These results support moving beyond a single “minimally speaking” label [57] toward the development of more individualized, strengths-based assessment and intervention approaches. Future studies should also examine whether underlying genetic etiologies contribute to these subgroup differences.

## Supplementary Material

This is a list of supplementary files associated with this preprint. Click to download.

- Additionalfile1.docx
- Additionalfile2.docx

## Figures and Tables

**Figure 1:**
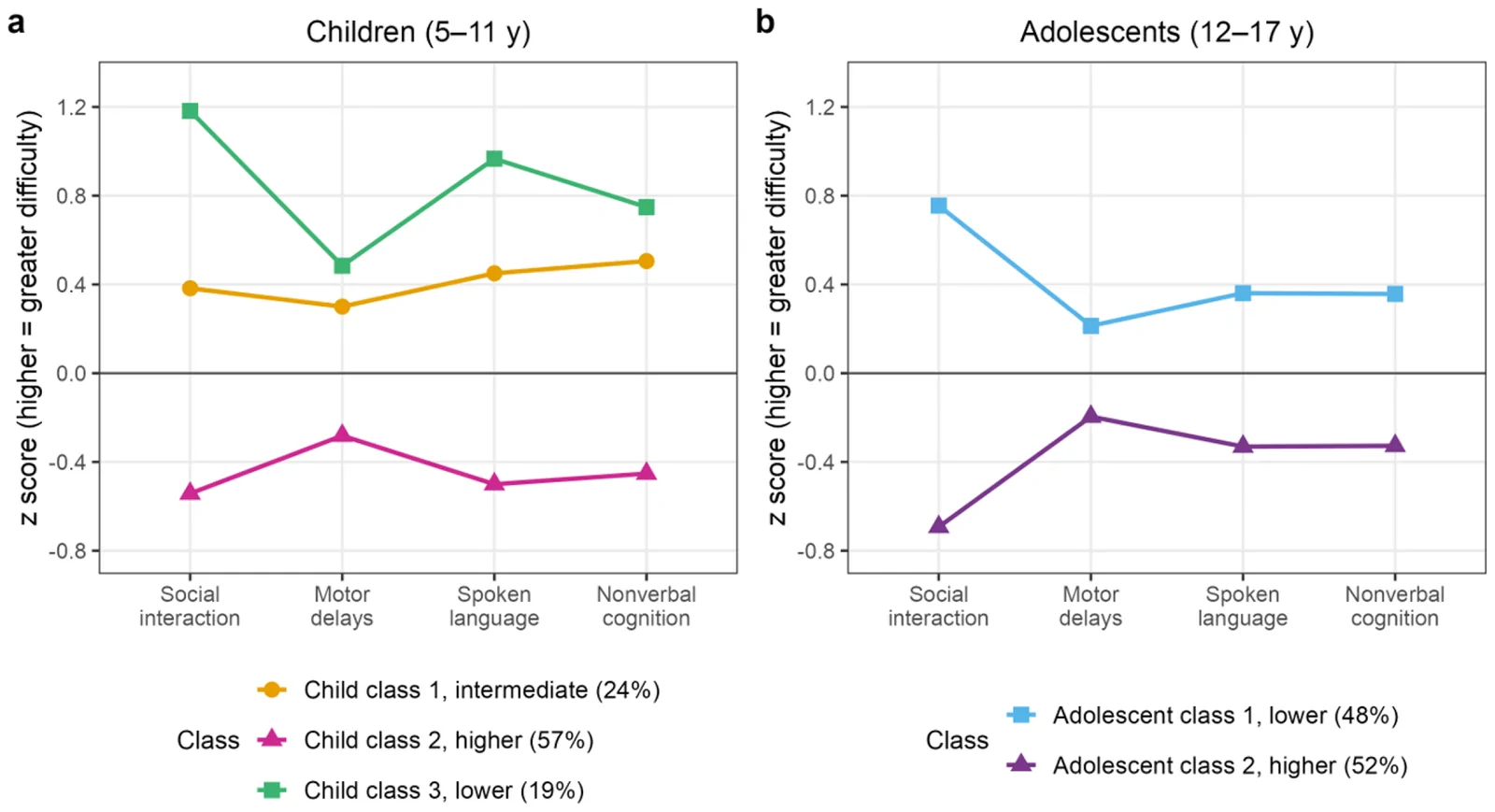
Latent class indicator profiles by age group. Mean within-age-group z scores for the four latent class indicators: social interaction, motor delays, spoken language, and nonverbal cognition. Panel a shows children aged 5–11 years. Panel b shows adolescents aged 12–17 years. In children, class 1 is intermediate-skill, class 2 higher-skill, and class 3 lower-skill. In adolescents, class 1 is lower-skill and class 2 higher-skill. The horizontal line marks the age-group mean (z = 0), and marker shapes indicate relative skill level: squares for lower, circles for intermediate, and triangles for higher skill. Labels are relative within age group. Higher z scores indicate greater difficulty: more difficulty with social interaction, more motor delays, less spoken language, and lower nonverbal cognition.

**Figure 2:**
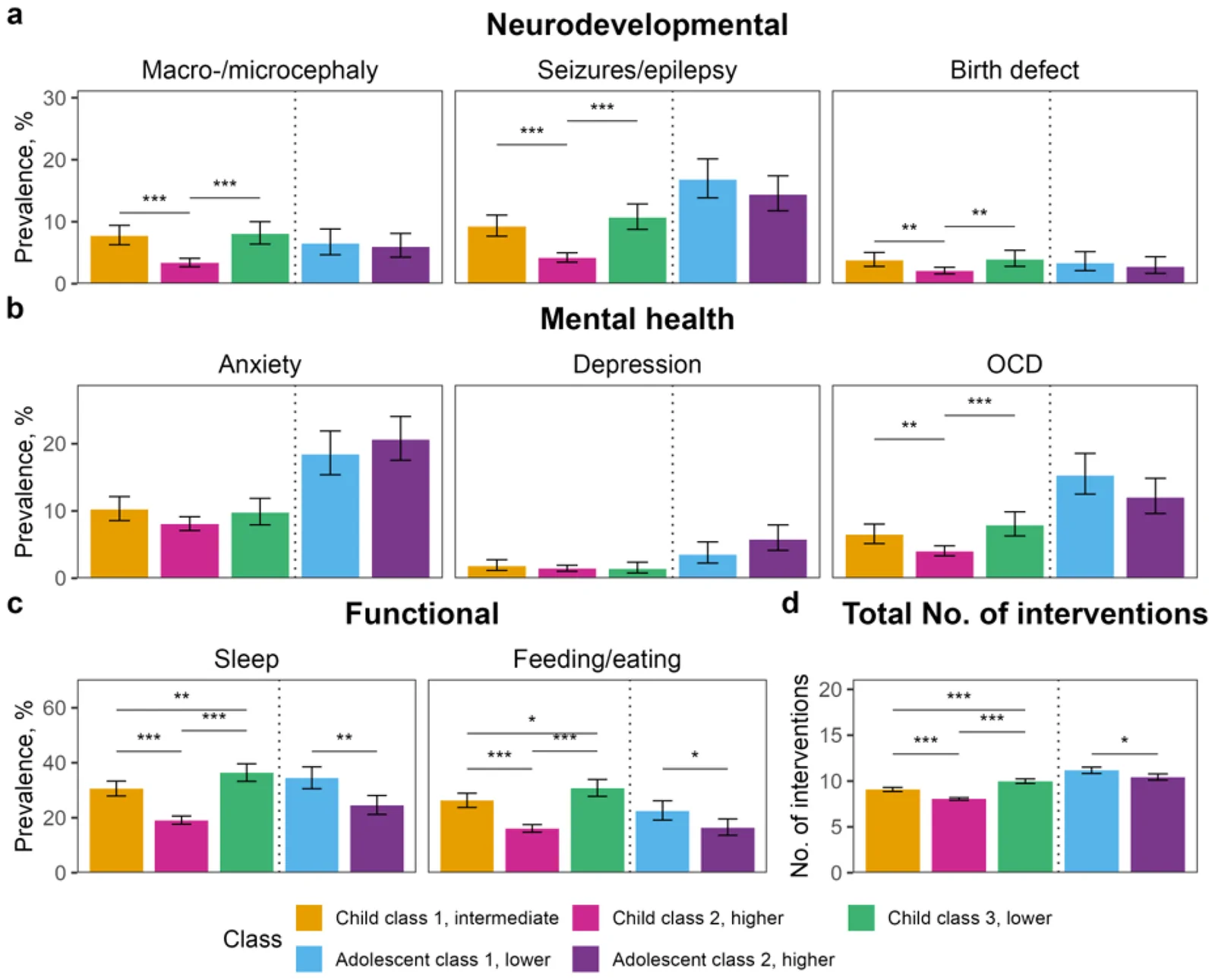
Prevalence of co-occurring conditions and service use by latent class. For conditions (a–c), bars indicate prevalence (%) with 95% Wilson score confidence intervals. For service use (d), bars indicate the mean total number of interventions with 95% confidence intervals. Child classes 1–3 are children aged 5–11 years and adolescent classes 1–2 are adolescents aged 12–17 years. The dotted line separates these age groups. Horizontal bars with asterisks denote significant differences between classes within an age group (Benjamini-Hochberg-adjusted q values: *q < .05; **q < .01; ***q < .001). For children, brackets show post hoc pairwise comparisons (Table S10). For adolescents, brackets show the FDR-adjusted omnibus comparisons between the two classes (Table 2). OCD, obsessive-compulsive disorder. Class labels are relative within age group: children, class 1 intermediate-skill, class 2 higher-skill, class 3 lower-skill; adolescents, class 1 lower-skill and class 2 higher-skill.

**Table 1:** Latent class indicator profiles by age group and class^a^

Characteristic	Children (5–11 y)				Adolescents	(12–17 y)	
	
	Intermediate-skill class 1 (n = 1116)	Higher-skill class 2 (n = 2692)	Lower-skill class 3 (n = 874)	P value^b^	Lower-skill class 1 (n = 543)	Higher-skill class 2 (n = 592)	P value^b^
**Age, y**				< .001			.12

Mean (SD)	7.6 (2.0)	7.1 (1.8)	8.1 (2.0)		14.6 (1.7)	14.4 (1.6)	

Median (IQR)	7.3 (5.9–9.1)	6.6 (5.6–8.1)	8.0 (6.4–9.7)		14.4 (13.1–16.0)	14.3 (13.2–15.6)	

**Everyday spoken language**				< .001			< .001

No words	365 (33)	95 (3.5)	554 (63)		200 (37)	78 (13)	

Single words	464 (42)	644 (24)	188 (22)		165 (30)	122 (21)	

Phrase speech	287 (26)	1953 (73)	132 (15)		178 (33)	392 (66)	

**Social interaction** ^c^				< .001			< .001

Mean (SD)	14.3 (2.3)	9.8 (4.3)	18.1 (1.3)		18.1 (1.4)	12.2 (3.6)	

Median (IQR)	14.0 (13.0–16.0)	10.0 (7.0–13.0)	18.0 (17.0–19.0)		18.0 (17.0–19.0)	13.0 (10.0–15.0)	

**Nonverbal cognition**				< .001			< .001

At or above age level	82 (7.3)	1003 (37)	34 (3.9)		20 (3.7)	91 (15)	

Slightly below age level	224 (20)	1027 (38)	63 (7.2)		33 (6.1)	163 (28)	

Significantly below age level	810 (73)	662 (25)	777 (89)		490 (90)	338 (57)	

**No. of motor delays**				< .001			< .001

0	328 (29)	1558 (58)	207 (24)		207 (38)	359 (61)	

1	371 (33)	687 (26)	289 (33)		132 (24)	102 (17)	

2	177 (16)	254 (9.4)	142 (16)		68 (13)	50 (8.4)	

3	111 (9.9)	107 (4.0)	85 (9.7)		67 (12)	42 (7.1)	

4	129 (12)	86 (3.2)	151 (17)		69 (13)	39 (6.6)	

IQR, interquartile range; SCQ-L, Social Communication Questionnaire-Lifetime; SD, standard deviation; y, years.

a Data are presented as No. (%) unless otherwise indicated.

b P values reflect comparisons within age group (χ^2^ or Fisher exact test for categorical variables, one-way analysis of means not assuming equal variances for continuous variables).

c Scores are for the 20-item SCQ-L social interaction domain (range 0–20, higher = greater difficulty). The total-score screening cutoff of 15 does not apply to this domain.

**Table 2:** Co-occurring conditions and service use by latent class and age group^a^

Condition	No. with data^b^	Children (5–11 y)					Adolescents (12–17 y)			
		Intermediate-skill class 1 (n = 1116)	Higher-skill class 2 (n = 2692)	Lower-skill class 3 (n = 874)	P value^c^	q value^d^	Lower-skill class 1 (n = 543)	Higher-skill class 2 (n = 592)	P value^c^	q value^d^
*Neurodevelopmental and medical conditions*										
Macro- or microcephaly	5802	86 (7.7)	90 (3.3)	70 (8.0)	< .001	< .001	35 (6.4)	35 (5.9)	.71	.71

Seizures or epilepsy	5802	103 (9.3)	112 (4.2)	93 (11)	< .001	< .001	91 (17)	85 (14)	.27	.40

Birth defect	5802	42 (3.8)	55 (2.0)	34 (3.9)	.001	.002	18 (3.3)	16 (2.7)	.55	.66

*Mental health and behavioral conditions*										
ADHD	5802	338 (30)	747 (28)	254 (29)	.27	.36	176 (32)	216 (37)	.14	.29

Anxiety	5802	114 (10)	217 (8.1)	85 (9.7)	.07	.10	100 (18)	122 (21)	.35	.46

Depression	5802	20 (1.8)	38 (1.4)	12 (1.4)	.64	.70	19 (3.5)	34 (5.8)	.07	.22

OCD	5802	72 (6.5)	108 (4.0)	69 (7.9)	< .001	< .001	83 (15)	71 (12)	.11	.26

Tics	5802	11 (1.0)	26 (1.0)	12 (1.4)	.58	.69	16 (2.9)	20 (3.4)	.67	.71

Conduct disorder or aggression	5802	68 (6.1)	149 (5.6)	51 (5.8)	.79	.79	56 (10)	48 (8.1)	.20	.35

*Functional conditions*										
Sleep disorder	5802	341 (31)	514 (19)	318 (36)	< .001	< .001	187 (34)	145 (25)	< .001	.003

Feeding or eating disorder	5802	293 (26)	433 (16)	269 (31)	< .001	< .001	122 (22)	97 (16)	.01	.04

*Service use*										
Total No. of interventions, mean (SD)	5817	9.1 (3.8)	8.1 (3.7)	10.0 (3.7)	< .001	< .001	11.2 (4.1)	10.4 (4.3)	.003	.02

ADHD, attention-deficit/hyperactivity disorder; FDR, false discovery rate; OCD, obsessive-compulsive disorder; SD, standard deviation; y, years.

a Data are presented as No. (%) unless otherwise indicated.

b Available-data denominators for co-occurring conditions were 1112, 2683, and 873 in child classes 1–3 and 543 and 591 in adolescent classes 1–2. Intervention counts were available for all members of each class.

c Comparisons were conducted across classes within each age group. P values are from the χ^2^ or Fisher exact test (categorical) and one-way analysis of means not assuming equal variances (Total No. of interventions).

d q values are Benjamini-Hochberg FDR-adjusted P values.

## Data Availability

The data analyzed in this study are available from the Simons Foundation Autism Research Initiative (SFARI) Base (https://base.sfari.org) to approved researchers, but restrictions apply. For example, to abide by the informed consent signed by SPARK participants and their families, the data are not publicly available and were used under approval from SFARI Base for the current study. Analytic code will be made publicly available on GitHub at the time of publication.

## Abbreviations

ADHD: attention-deficit/hyperactivity disorder
BHQ: Background and History Questionnaire-Child/Dependent
BIC: Bayesian information criterion
CI: confidence interval
DSM-5: Diagnostic and Statistical Manual of Mental Disorders, Fifth Edition
EVI: equal-volume, varying-shape covariance parameterization
FDR: false discovery rate
ICL: integrated completed likelihood
IQR: interquartile range
OCD: obsessive-compulsive disorder
SCQ-L: Social Communication Questionnaire-Lifetime
SD: standard deviation
SFARI: Simons Foundation Autism Research Initiative
SPARK: Simons Powering Autism Research for Knowledge
STROBE: Strengthening the Reporting of Observational Studies in Epidemiology
WCG IRB: Western Institutional Review Board–Copernicus Group
